# Long-read plasmid sequencing strategy for evaluating intrinsic instability of tandem gene arrays

**DOI:** 10.17912/micropub.biology.001582

**Published:** 2025-05-08

**Authors:** Hiroaki Takesue, Satoshi Okada, Takashi Ito

**Affiliations:** 1 Department of Biochemistry, Kyushu University Graduate School of Medical Sciences, Fukuoka, Japan

## Abstract

Tandem gene arrays are inherently unstable, particularly in recombination-prone organisms such as the budding yeast *Saccharomyces cerevisiae* . However, understanding the nature of this instability—how frequently and to what extent the target array contracts or expands within the genome—remains challenging. As a surrogate approach to this goal, we propose using nanopore long-read sequencing to directly determine the length distribution of a target gene array cloned onto a centromeric plasmid functioning as an artificial chromosome. This strategy will not only allow us to assess the intrinsic instability of the array but also help identify factors that may influence its stability.

**Figure 1. Distribution of CUP1 copy numbers on centromeric plasmids in budding yeast:**
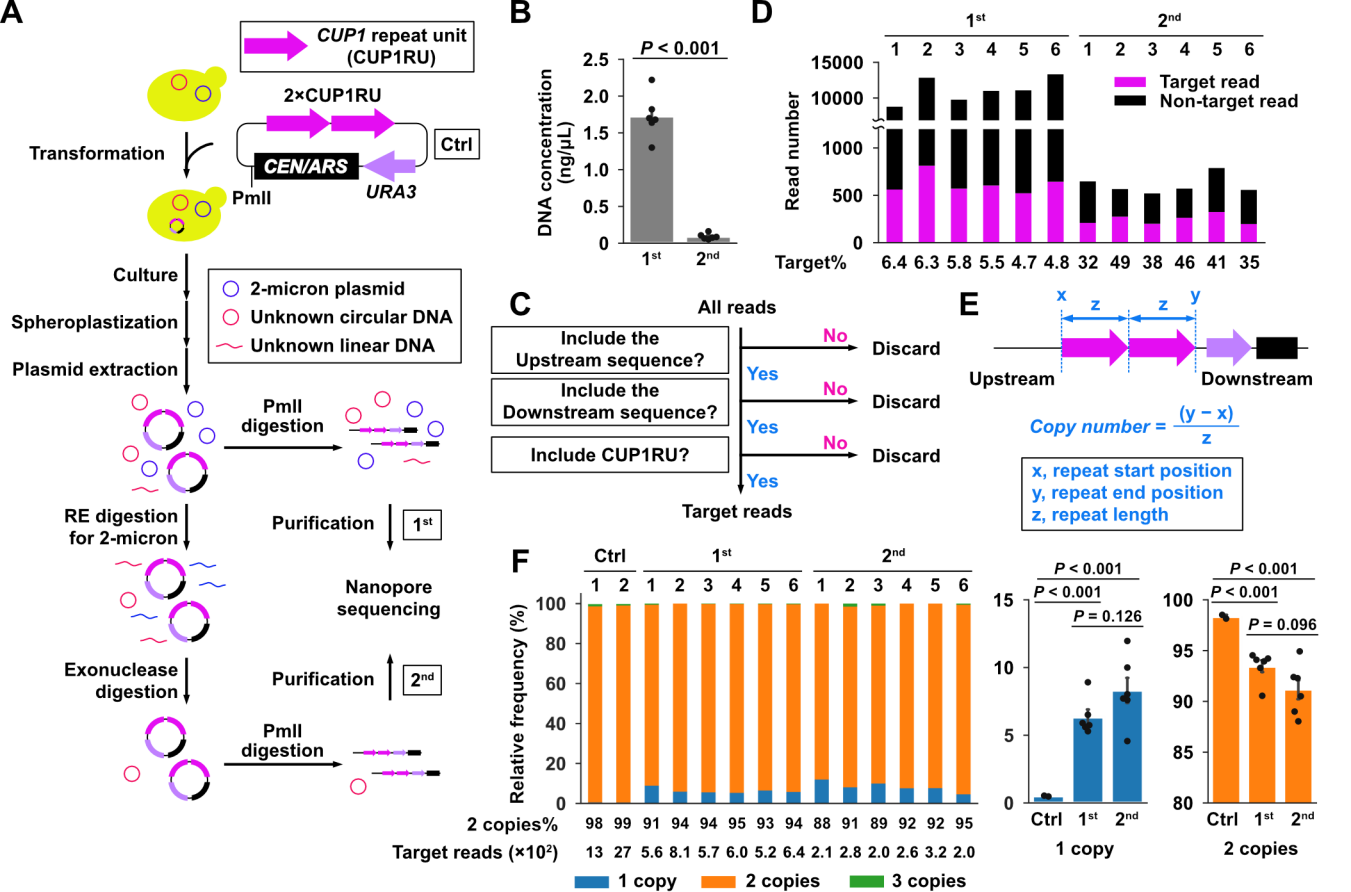
( **A** ) Flowchart illustrating the process from the transformation of the test plasmid YCpURA-2×CUP1RU in budding yeast to library preparation for nanopore sequencing. Ctrl, the control sample or the plasmid used for transformation; RE, restriction enzyme; 1 ^st^ and 2 ^nd^ , DNA samples prepared using the first and second protocols, respectively. ( **B** ) DNA concentrations after purification using the first and second protocols shown in (A). Data are represented as mean ± standard deviation ( *n* = 6 biological replicates). Statistical significance was examined using a *­t­* -test. ( **C** ) Flowchart depicting the strategy to select target reads from all nanopore reads. ( **D** ) Numbers of the target and non-target nanopore reads obtained using the Flongle flowcell. ( **E** ) Conceptual diagram of the formula used to calculate the copy number from the target reads selected in (C). ( **F** ) Distribution of *CUP1* copy numbers in each sample. The percentages of one- and two-copy *CUP1* repeat unit (CUP1RU) in each group are represented as mean ± standard deviation ( *n* = 2 or 6 biological replicates). Statistical significance was examined using a *­t­* -test.

## Description

Tandem gene duplication has played a central role in evolution, serving as a source of genetic diversity across various eukaryotic species, including pathogenic copy number variations in humans. It can lead to further recombination events between the repeat units, resulting in either expansion into a tandem gene array or contraction to a single-copy gene. Consequently, tandem gene arrays are inherently unstable, with the degree of instability varying among different arrays, presumably influenced by the nature of the repeat unit and the surrounding genomic environment.

To understand these factors, accurately assessing the frequency and extent of destabilization is crucial. While traditional Southern blot hybridization effectively determines the distribution of array lengths, it has limitations in detecting low-frequency variants. Long-read sequencing emerges as a promising alternative for this purpose. To maximize its potential at a reasonable cost, enriching target gene arrays through hybridization or Cas9-mediated excision is ideal; however, these approaches can be technically demanding.

Therefore, we propose a simple plasmid-based method as a surrogate assay for investigating the intrinsic instability of tandem gene arrays, leveraging cost-effective long-read sequencing. We used the budding yeast *CUP1* array, which encodes a copper metallothionein, as our model system to evaluate instability.

We constructed the minimum *CUP1* array, consisting of two 2-kb repeat units expected to be the most stable, on a centromeric plasmid marked with *URA3* ( Figure 1A, YCpURA-2×CUP1RU). Cells transformed with YCpURA-2×CUP1RU were grown overnight in synthetic complete medium lacking uracil (SC−Ura) before plasmid extraction. We used two protocols to process the extracted plasmid DNA, which included both YCpURA-2×CUP1RU and the endogenous 2-micron plasmid. In the first protocol, the plasmid DNA was treated with the restriction enzyme PmlI, which cleaves YCpURA-2×CUP1RU but not the 2-micron plasmid ( Figure 1A, 1 ^st^ ), resulting in 1.73 ± 0.30 ng of purified DNA from half of an overnight 2-mL culture (~2.0 × 10 ^8^ cells) ( Figure 1B, 1 ^st^ ). In the second protocol, the plasmid DNA was treated with BsoBI and SphI to linearize the 2-micron plasmid without affecting YCpURA-2×CUP1RU, followed by exonuclease V (RecBCD) treatment to degrade the linear DNA. After heat inactivation of exonuclease V, PmlI digestion was performed to linearize YCpURA-2×CUP1RU ( Figure 1A, 2 ^nd^ ), resulting in 0.09 ± 0.04 ng of purified DNA from the other half of the culture ( Figure 1B, 2 ^nd^ ).

The 1 ^st^ and 2 ^nd^ samples purified from the yeast cells were subjected to Nanopore sequencing using the Native Barcoding Kit along with the Ctrl sample or the test plasmid prepared from *E. coli* for yeast transformation. The obtained reads were basecalled in super accuracy mode. Target reads containing both the vector and the *CUP1* array were extracted from the obtained reads ( Figure 1C ). The 1 ^st^ and 2 ^nd^ samples contained the target reads at frequencies of 5.6% and 40.3%, respectively, demonstrating substantial enrichment of the target plasmid by eliminating the 2-micron plasmid and other linear DNAs ( Figure 1D ). The non-target reads in the 1 ^st^ sample were predominantly derived from ribosomal and mitochondrial DNAs, whereas those in the 2 ^nd^ sample exhibited no dominant DNA species. Given that the average length of non-target DNA fragments in the samples is similar to that of the target plasmid, we estimate the amounts of the target plasmid to be 0.096 ng (= 1.73 ng × 0.056) in the 1 ^st^ sample and 0.036 ng (= 0.09 ng × 0.403) in the 2 ^nd^ sample. These results suggest that the additional steps in the second protocol likely reduced the yield by 62.5% compared to the first protocol.

We then calculated the *CUP1* copy number in the target reads using the formula shown in Figure 1E . The majority of reads contained two copies, while those with one copy (contraction) and three copies (expansion) accounted for an average of 7.32% and 0.36% of the reads, respectively ( Figure 1F ). Compared to the Ctrl sample obtained from *E. coli* , we observed a significant increase in the percentage of single-copy *CUP1RU* and a corresponding decrease in two-unit *CUP1* arrays, regardless of the protocols used ( Figure 1F ). These results suggest that contraction occurs at a substantial frequency, even in the two-unit *CUP1* array within yeast. Importantly, the difference in contraction frequencies between the 1 ^st^ and 2 ^nd^ samples was not statistically significant. Moreover, according to Oxford Nanopore Technologies' guidelines, maintaining most pores in an active sequencing state is crucial for preserving pore quality. Therefore, removing non-target DNA from extracted plasmid DNA samples is unnecessary as long as the number of samples is not excessively high.

The plasmid-based assay described above would provide a versatile method for identifying factors affecting tandem gene arrays, as it can reveal the distribution of array lengths. For the *CUP1* array, it can be readily applied to examine various conditions, including the presence of copper in the medium and the absence of genes potentially affecting array stability. It would also facilitate the evaluation of the effects of the dCas9/nCas9-mediated interrogations we have developed for the targeted contraction and expansion of tandem gene array (Doi et al. 2021; Takesue et al. 2025).

## Methods

*Plasmid cloning*

A centromeric plasmid containing two tandemly repeated copies of *CUP1RU* and the *URA3* marker gene (YCpURA-2×CUP1RU) was constructed by seamless cloning using Golden Gate Assembly (New England Biolabs) and then transformed into *E. coli* using the Champion™ DH5α High competent cells (SMOBIO).

*Yeast strains*

The yeast strain used in this study was YIT12685 derived from BY4741 ( *MAT* **a** *his3* Δ1 *leu2* Δ0 *met15* Δ0 *ura3* Δ0) (Brachmann et al. 1998). This study used standard culture media and genetic methods(Guthrie and Fink 1991). The yeast strain was transformed with the YCpURA-2×CUP1RU plasmid using the standard lithium acetate transformation protocol. Yeast cells were grown at 30°C overnight in 2 mL of the SC−Ura medium supplemented with 2% glucose.

*Plasmid extraction*

Yeast cells were washed with phosphate-buffered saline (PBS). After centrifugation, pellets were resuspended with spheroplast buffer [1 M sorbitol, 1× PBS pH 7.4, and 0.1 M EDTA] and 2.5 U Zymolyase (Zymo Research), and cell walls were digested at 30°C for 20 min, inverting every 5 min. After centrifugation for 3 min at 300× *g* , plasmid DNA was extracted from the spheroplast pellet using FavorPrep plasmid extraction mini kit (FAVORGEN). DNA concentration was calculated by Qubit 3.0 Fluorometer with Qubit dsDNA HS Assay System (Thermo Fisher Scientific). Non-target DNA was digested with restriction enzymes BsoBI (New England Biolabs), SphI-HF (New England Biolabs), and the exonuclease RecBCD (New England Biolabs), and the enzymes were denaturated at 80°C for 20 min. Plasmid DNA was linearized with the restriction enzyme PmlI (New England Biolabs) and purified with 0.4× AMPure XP (Beckman Coulter).

*Nanopore sequencing*

DNA libraries were prepared using the native barcoding kit SQK-NBD114 (Oxford Nanopore Technologies) according to the manufacturer’s instructions. The library was sequenced with the flowcell FLO-FLG114 R10.4.1 using the MinION sequencer (Oxford Nanopore Technologies). MinKNOW software was used to control the MinION device. The run time was set to 24 h. Base calling was performed using Dorado v0.7.3. The assessment of sequencing data was performed using NanoPlot (De Coster et al. 2018).

*Copy number estimation from nanopore reads*

We used nanopore sequencing data in FASTQ format and mapped reads to the S288c reference genome (version R64-2-1, http://sgd-archive.yeastgenome.org/sequence/S288C_reference/genome_releases/S288C_reference_genome_R64-2-1_20150113.tgz) using SAMtools (Danecek et al. 2021) and BEDtools (Quinlan et al. 2010). We then calculated normalized read count of each nucleotide using Bedgraph_norm_ratio.py (https://doi.org/10.5281/zenodo.11515696). Data were visualized with the IGV (Robinson et al. 2011). To eliminate the effect of read clipping and achieve a more accurate estimation of repeat unit number, we collected all reads containing the repeat unit using minialign (https://github.com/ocxtal/minialign). We then used its reference sequence as a query in a BLAST search (Altschul et al. 1990) against the collected reads and estimated the copy number based on the number of BLAST hits.

*Generative AI and AI-assisted technologies*

During the preparation of this work, the authors used GPT-4o to improve the readability of certain sentences. After using this tool/service, the authors reviewed and edited the content as needed and take full responsibility for the content of the publication.

## Reagents

The yeast strain used in this study.

**Table:** 

Strain	Genotype	Available from
YIT12685	*met15* Δ:: *pCUP2-yGEV-tADH1* *ho* Δ:: *pGAL1-nCas9(D10A)-tADH1*	Ito lab

The plasmid used in this study.

**Table:** 

Plasmid	Genotype	Description
34-29	YCpURA-2×CUP1RU	A centromeric plasmid bearing two tandemly repeated copies of *CUP1RU* and the *URA3* marker gene
